# Does cranberry extract reduce antibiotic use for symptoms of acute uncomplicated urinary tract infections (CUTI)? Protocol for a feasibility study

**DOI:** 10.1186/s13063-019-3860-z

**Published:** 2019-12-23

**Authors:** Oghenekome Gbinigie, Julie Allen, Anne-Marie Boylan, Alastair Hay, Carl Heneghan, Michael Moore, Nicola Williams, Chris Butler

**Affiliations:** 1 Nuffield Department of Primary Care Health Sciences, University of Oxford, Radcliffe Observatory Quarter, Woodstock Road, Oxford, OX2 6GG UK; 2 Centre for Academic Primary Care, University of Bristol, Bristol, BS8 2PS UK; 3 Primary Care and Population Sciences, University of Southampton, Southampton, SO16 5ST UK

**Keywords:** Urinary tract infection, Cranberry, *Vaccinium macrocarpon*

## Abstract

**Background:**

Consultations in primary care for symptoms of urinary tract infections (UTIs) are common and patients are frequently treated with antibiotics. Given increasing antimicrobial resistance, there has been interest in non-antibiotic treatment options for common infections. One such option is the use of cranberry extract to treat symptoms attributable to UTIs.

**Methods:**

A target of 45 women consulting in primary care, with symptoms suggestive of an uncomplicated UTI for whom the practitioner would normally prescribe antibiotics, will be randomised to receive one of three treatment approaches: (1) immediate prescription for antibiotics; (2) immediate prescription for antibiotics plus a 7-day course of cranberry capsules and (3) cranberry capsules plus a delayed prescription for antibiotics to be used in case their symptoms do not get better, or get worse. Follow-up will be by daily rating of symptoms and recording of treatments used for 2 weeks in an online symptom diary. Interviews will be conducted with around 10–15 study participants, as well as with around 10–15 women who have experienced a UTI but have not been approached to take part in the study. Both groups will be asked about their experience of having a UTI, their thoughts on non-antibiotic treatments for UTIs and their thoughts on, or experience of, the feasibility trial. The primary objective is to assess the feasibility of undertaking a full trial in primary care of the effectiveness of cranberry extract to reduce antibiotic use for symptoms of acute uncomplicated UTI. The secondary objective is to conduct a preliminary assessment of the extent to which cranberry might reduce antibiotic use and symptom burden.

**Discussion:**

This feasibility study with embedded interviews will inform the planning and sample size calculation of an adequately powered trial to definitively determine whether cranberry helps to alleviate the symptoms of acute uncomplicated UTIs in women and whether it can safely reduce antibiotic use.

**Trial registration:**

ISRCTN registry, ID: 10399299. Registered on 24 January 2019.

## Background

Symptoms of uncomplicated urinary tract infection (UTI) are a frequent presentation in primary care and are usually treated with antibiotics [1]. Antibiotic prescribing in primary care is associated with increasing antibiotic resistance (AMR) [2]. Amid concerns that we are entering a ‘post-antibiotic era’ [3], there has been growing interest in the use of non-antibiotic treatments for common, presumed bacterial infections, such as UTIs, in order to reduce AMR.

Gagyor et al. [4] found that women with acute UTIs treated with ibuprofen used fewer antibiotics over 28 days compared to women who were treated with fosfomycin (Incidence rate reduction of 66.5% (95% CI 58.8% to 74.4%, *p* < 0.001)). However, women treated with ibuprofen had a greater symptom burden compared to those treated with fosfomycin (mean difference in symptom burden between groups 5.3% (95% CI 3.5 to 7%, *p* < 0.001)) and a non-significant increase in the number of cases of pyelonephritis (mean difference 1.7% (95% CI − 0.3 to 3.6, *p* = 0.12)). Kronenberg et al. [5] similarly found that women treated with diclofenac for an acute UTI used fewer antibiotics within 30 days compared with those treated with norfloxacin (risk difference 37% (95% CI 28 to 46%, *p* < 0.001 for superiority)). However, norfloxacin treatment led to earlier resolution of symptoms (risk difference in symptom resolution at day of 27% (95% CI 15 to 38%, *p* < 0.001 for superiority) and fewer cases of pyelonephritis (risk difference 5 (95% CI 1 to 8, *p* = 0.031)), compared with diclofenac. A further study comparing ibuprofen with pivmecillinam for acute UTI in women [6] found that more people treated with ibuprofen required secondary treatment with antibiotics by day 28 (adjusted risk difference 36% (95% CI 27 to 44%)) and more women treated with ibuprofen had episodes of pyelonephritis (adjusted risk difference 4% (95% CI 1 to 8%)). Fewer people treated with ibuprofen were symptom free by day 4 (adjusted risk difference 35% (90% CI 27 to 43%)).

A placebo-controlled trial assessing the ability of a herbal remedy, Uva-ursi, with or without advice to take ibuprofen to reduce antibiotic use for uncomplicated UTI found that there was no significant improvement in frequency symptoms between those taking Uva-ursi and placebo (− 0.06 (95% CI − 0.33 to 0.21; *p* = 0.661)), nor between those taking ibuprofen and those not advised to take ibuprofen (− 0.01 (95% CI − 0.27 to 0.26; *p* = 0.951)) [7]. Antibiotic consumption was not significantly reduced with Uva-Ursi (OR 0.59 (95% CI 0.22 to 1.58; *p* = 0.293)), but was reduced with ibuprofen (OR 0.27 (95% CI 0.10 to 0.72; *p* = 0.009)). There were no episodes of pyelonephritis.

Another non-antibiotic candidate treatment for uncomplicated UTIs is cranberry preparations (*Vaccinium macrocarpon*). Up to 27% of women already use cranberry to help treat UTIs, despite the lack of robust trial evidence for its benefit [1]. The mechanism of action by which cranberry might treat UTIs is unclear. The active ingredient may be proanthocyanidins (PAC) with Type A linkages. PAC with Type A linkages is thought to prevent *Escherichia coli* (*E. coli*) from binding to the uroepithelial lining [8], therefore preventing *E. coli* from causing and sustaining a UTI. Trials have assessed the use of cranberry products to prevent *recurrent* UTIs. The most recent Cochrane review on the topic found that cranberry products did not prevent recurrent UTIs in women any more than placebo or no treatment (RR 0.86, 95% CI 0.71 to 1.04) [9]. However, the reviewers highlighted a lack of standardisation of the amount of active ingredient consumed by participants of studies [9] and noted withdrawals of up to 55%, largely due to having to consume large volumes of cranberry juice for prolonged periods on a daily basis [9].

Thus far, few studies have assessed the potential benefit of cranberry in *treating* symptoms of an acute, uncomplicated UTI, or indeed assessed whether cranberry might have a synergistic effect when combined with antibiotics [10–12]. It is possible that through preventing *E. coli* from binding to the uroepithelial lining, cranberry may enhance bactericidal effects of urinary antibiotics. If cranberry extract safely and effectively treats uncomplicated UTIs or acts synergistically with antibiotics, this could substantially help to reduce the overall antibiotic exposure. A well-designed and adequately powered study is required to assess this. However, given that we have not been able to identify adequate published data relevant for planning such a study, we set out to conduct a feasibility study to facilitate planning such an effectiveness randomised controlled trial (RCT).

## Methods

### Aim

#### Primary objective

To assess the feasibility of undertaking a trial in primary care of the effectiveness of cranberry extract for symptoms of acute uncomplicated UTI.

#### Secondary objective

Preliminary assessment of the extent to which cranberry supplementation affects the number of antibiotic courses consumed for acute uncomplicated UTI, and impact on symptom burden.

### Design and setting

This will be a three armed, open-label, randomised clinical trial to assess the feasibility of conducting a RCT comparing the effect of immediate antibiotics, immediate antibiotics and cranberry capsules, and immediate cranberry capsules with a delayed prescription for antibiotics, on antibiotic consumption and symptom burden in women with symptoms of acute uncomplicated UTI.

Women presenting with symptoms of uncomplicated UTI to participating general practices in Oxfordshire will be invited to participate. The feasibility study requires a single consultation with a healthcare practitioner/researcher for each participant, keeping a symptom diary for up to 14 days, and email ± telephone follow-up at day 14.

### Eligibility

Participants suitable for inclusion will be women aged 18 years and above who are willing and able to give informed consent for participation in the study. Women will be eligible if they consult a participating GP practice with urinary symptoms suggestive of acute, uncomplicated lower UTI (namely dysuria, urgency, frequency, polyuria/nocturia, haematuria and/or suprapubic pain), which the GP or healthcare practitioner consider should be treated with an immediate prescription of antibiotics. Symptom duration should be 6 days or less, and the potential participant must be willing to receive either an immediate or delayed antibiotic prescription.

Exclusions will be: cranberry allergy; having taken an antibiotic in the past 7 days; current regular cranberry product consumption (5 or more days a week); known or suspected pregnancy; breastfeeding; taking anti-coagulants (e.g. warfarin) or anti-platelet agents; unable to obtain a clean-catch/midstream urine sample (e.g. because of incontinence); in-dwelling catheter; receiving end-of-life care/palliative care; known underlying structural urological abnormalities; previous urological surgery; immunosuppressed (e.g. active cancer (excluding localised skin cancer); receiving chemotherapy; taking regular high-dose orally administered steroids (> 5 mg/day), HIV infection); diabetes mellitus treated with insulin; signs of clinically suspected upper UTI/pyelonephritis; inability to complete symptom diary accurately (e.g. dementia or psychosis); already involved in an interventional research study on UTI; unable to access Internet/email for the next 2 weeks; unable to decide on the same day that they contacted a primary care provider whether they would like to participate.

### Recruitment and randomisation

**Fig. 1:**
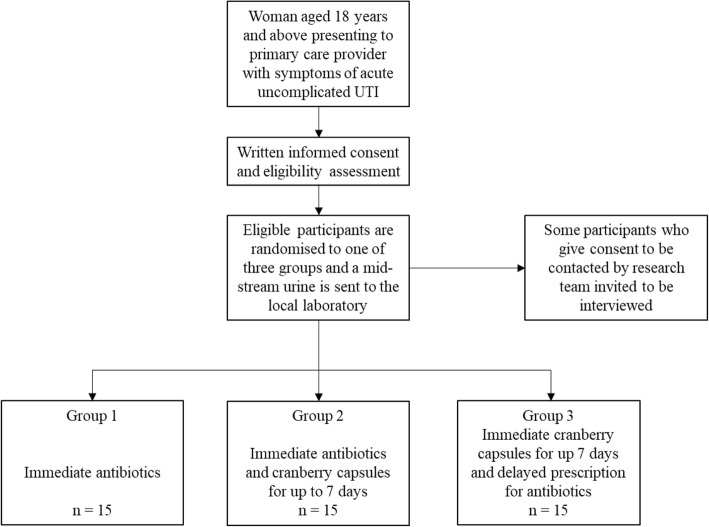
Recruitment and randomisation

We aim to recruit 45 participants from primary care sites in Oxfordshire (see Fig. 1). As this is a feasibility study to inform the design and delivery plans for an adequately powered multi-centre RCT, we believe that a sample size of approximately 45 will provide sufficient data to determine whether the current study design works well. With a sample size of 45, we would be able to estimate a loss-to-follow-up rate of 20% with a confidence interval of 9.6 to 34.6%. Given that UTI consultations account for 3% of all GP consultations [13], we anticipate being able to recruit at least two participants per month per GP practice. This would equate to 12 participants from one GP practice over 6 months. We hope to recruit from a minimum of four GP practices.

Recruitment will be opportunistic; when a member of the primary care centre team is in contact with a potential participant who may be suitable for inclusion, the potential participant will be approached to take part in the study. This could be in a GP practice or during a telephone consultation. Eligible participants who are interested in participating in the study will be given a Participant Information Leaflet (PIL). The PIL will give full details of the feasibility study. Participants who are recruited by telephone will be given a verbal summary of the key information contained in the PIL. If interested in participating, the women will be invited to attend the primary care centre the same day and will receive a written version of the PIL. Written, informed consent will be obtained by a healthcare professional or a suitably qualified/trained person before any study procedures begin (see Additional files 1 and 2 for the study PIL and Informed Consent Form (ICF)). Study posters will also be placed in participating primary care centres to increase potential participant awareness of the study.

Research Data Capture (REDCap) and Sentry electronic databases will be used to register participants to the feasibility study. REDCap is a secure, password-protected, web-based system, which will be used to store the electronic Case Report Forms (CRFs)/information from the symptom diaries. REDCap will store participant ID numbers, but no participant-identifiable information. REDCap will incorporate data-entry and validation rules to reduce data-entry errors, and management functions to facilitate auditing and data-quality assurance. Sentry is a secure, password-protected and encrypted database; this will be used to store participant-identifiable information and will have restricted access.

Participants will be randomly assigned to one of the three groups in the study in a 1:1:1 ratio. The randomisation sequence will be generated by a statistician based at Nuffield Department for Primary Care Health Sciences (NDPCHS), Oxford, using computer-generated block randomisation with variable block size. The randomisation code will be accessible only by the study statistician. REDCap software will randomise participants electronically through the click of a button by the recruiter during participant registration. Neither the recruiter nor the participant will know to which group the participant has been randomly allocated in advance of this step.

### Study interventions

Participants will be randomly assigned to one of three groups:

- *Group 1 – Immediate prescription of first-line recommended antibiotics alone*

Recruiters will advise participants that they will be free to use additional medicine/symptomatic treatments (apart from cranberry juice/extract) to control symptoms at their discretion. This is the ‘control’ arm.

- *Group 2 – Immediate prescription of first-line recommended antibiotics with the addition of cranberry capsules (provided by the research team) for up to 7 days*

The responsible clinician will give participants an antibiotic prescription and, in addition, cranberry (Redicran) capsules containing 60 mg of cranberry extract (Anthocran) and 18 mg of PAC. Through ex-vivo experimentation, Howell et al. [14] found that peak urinary anti-adhesion activity of PAC occurred at 6 h, and standardised the optimal amount of PAC to consume at 36 mg twice a day. Participants will, therefore, be advised by recruiters to take two cranberry capsules twice a day, 12 h apart, until they are free of symptoms, but up to a maximum of 7 days. Cranberry capsules will be supplied free of charge by Indena S.p.A; this company will have no other involvement in the study (the design, data collection, analysis or interpretation of findings). Indena S.p.A manufactures the cranberry extract (Anthocran), which is used by a different company (Oniria S.r.l.) to produce the cranberry (Redicran) capsules in compliance with the applicable European Union legislation. These capsules are available on the Italian market.

Participants can use additional medicine/symptomatic treatments to control symptoms at their discretion. Data from this arm in an adequately powered study would assess whether cranberry in addition to antibiotics helps to reduce symptom burden.

- *Group 3 – Delayed prescription of first-line recommended antibiotics and immediate cranberry capsules (provided by the study) for up to 7 days*

Participants will receive cranberry capsules (Redicran) and will be advised to take two capsules twice a day, 12 h apart, until they are free of symptoms, but up to a maximum of 7 days. Participants will also receive a delayed prescription for the antibiotic that the healthcare practitioner would ordinarily have prescribed for immediate use. Participants will be advised by the responsible clinician to start the antibiotics if their symptoms worsen or fail to improve after 3–5 days. Participants can use additional medicine/symptomatic treatments to control symptoms at their discretion. Data from this arm in an adequately powered study would help to determine whether antibiotic use can be safely reduced through cranberry consumption.

If not already done as part of routine clinical care, all participants will be asked to provide a mid-stream urine (MSU) sample to be sent to the GP practice’s local laboratory for microscopy, culture and sensitivity.

### Assessments and follow-up

Participants will be asked to complete an electronic symptom diary containing three sections over a period of 2 weeks [7, 15] (see Additional file 3 for the paper version of the symptom diary).

#### Section 1 of the symptom diary

On the day of recruitment, participants will be asked to enter baseline information. This will include documenting whether they have a history of UTIs, use of over-the-counter treatments that have been tried before seeing the GP for their current UTI symptoms (e.g. paracetamol, ibuprofen or uvacin).

#### Section 2 of the symptom diary

Participants will rate the severity of their symptoms each day for up to 2 weeks (depending on the duration of their symptoms) on a scale of 0–50. This is an adaptation of the 6-point Likert scale developed by Watson et al. (2001) [16].

- 0 = Normal/not affected
- 1–9 = Very little problem
- 10–19 = Slight problem
- 20–29 = Moderately bad
- 30–39 = Bad
- 40–49 = Very bad
- 50 = As bad as it could be

Section 2 of the symptom diary will also allow participants to enter any treatments/medications that they take each day. Participants will be emailed a link each day to complete the diary online for that day.

#### Section 3 of the symptom diary

In the final section of the symptom diary (day 14), participants will complete a final set of questions, including questions on adverse effects suffered as a result of participation in the study, how easy/difficult they found it to complete the electronic diary and how acceptable they found the study.

Participants will be emailed to remind them to complete Section 3 of the electronic symptom diary and/or telephoned to encourage completion of the electronic symptom diary and to obtain a minimal dataset if the diary has not been adequately completed. Participants who complete the symptom diary will receive a £10 voucher in recognition of their contribution to the study. Each participant will have the right to withdraw from the study at any time without giving a reason. Any data collected from that participant up until withdrawal will be kept.

#### Notes’ review at 1 month

**Fig. 2:**
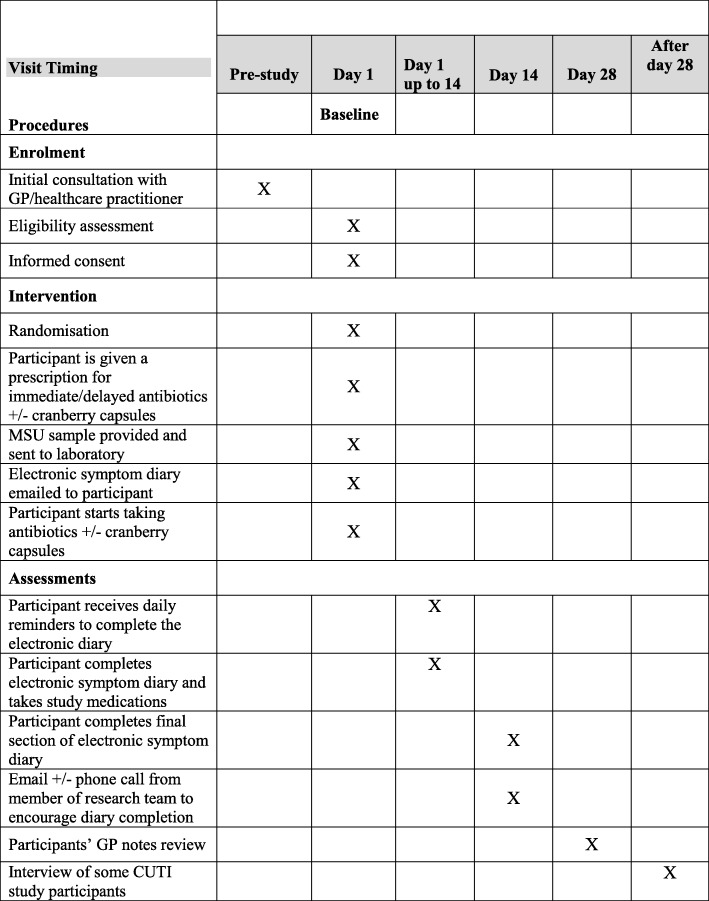
Standard Protocol Items: Recommendations for Interventional Trials (SPIRIT) Figure showing schedule of procedures

Participants’ GP records will be reviewed to record any attendances to the GP practice, hospital or out-of-hours facility within 28 days of the baseline assessment that are believed to be related to the original UTI episode. The result of the original urine culture will be recorded on REDCap; a positive growth will be defined as a growth of an organism greater than 10^5^ CFU/ml. The Standard Protocol Items: Recommendations for Interventional Trials (SPIRIT) Figure shows the schedule for enrolment, intervention,s and assessments (see Fig. 2 and Additional file 4).

### Safety reporting

#### Adverse events

An adverse event (AE) is any untoward medical occurrence that does not necessarily have a causal relationship with the medicinal product administered. Participants in group 3 of the feasibility study will not receive immediate antibiotics and may, therefore, be at higher risk of developing an upper UTI. In the event of a participant developing upper urinary tract symptoms, they will be advised (by the consenting clinician and information in the study pack) to urgently contact a medical professional. It will be emphasised in study materials and when training participating clinicians that a 7-day course (rather than a 3-day course) of antibiotics is required and that the first-line recommended antibiotic may be different to the antibiotic that the participant has been prescribed. Details of the symptoms of upper UTI and actions to take should they develop will be included within the study materials. All episodes of upper UTI that develop up to 28 days post randomisation will be recorded.

Discontinuation of cranberry capsules will occur if a participant has an adverse reaction to the capsules, becomes pregnant, or develops a concurrent illness that the Principal Investigator (PI) or Chief Investigator (CI) feels precludes ongoing study involvement.

AEs will be reported in four ways: (1) Participants may contact the study team using the contact details contained within the study pack. We will not provide 24-h cover as this is an open-label, low-risk study, (2) Participants may record details of AEs within the symptom diary, (3) AEs may be reported to GP practices and (4) AEs may be picked up during the participants’ notes’ review at 1 month.

#### Serious adverse events

A serious adverse event (SAE) is any untoward medical occurrence that results in death, is life-threatening, requires inpatient hospitalisation or prolongation of existing hospitalisation or results in persistent or significant disability/incapacity. Other ‘important medical events’ may also be considered serious if they jeopardise the participant or require an intervention to prevent one of the above consequences. An SAE occurring to a participant will be reported to the Research Ethics Committee (REC) that gave a favourable opinion of the study where in the opinion of the CI the event was ‘related’ (resulted from administration of any of the research procedures) and ‘unexpected’ in relation to those procedures. Reports of related and unexpected SAEs will be submitted within 15 working days of the CI becoming aware of the event.

We are not aware of any serious/common side effects that are associated with consuming cranberry (Redicran) capsules.

### Monitoring

The Trial Steering Committee (TSC) will provide overall supervision for the study. As this is anticipated to be a low-risk interventional study, there will not be a separate Data Monitoring Committee (DMC). The TSC will also assume the role of the DMC in safeguarding the interests of study participants and monitoring the overall conduct of the study. The TSC will be kept informed of substantial protocol amendments.

Direct access will be granted to authorised representatives from the sponsor (University of Oxford) and host institution (Nuffield Department of Primary Care Health Sciences, Oxford) for monitoring and/or audit of the study to ensure compliance with regulations. The University of Oxford will have access to the final study dataset. Access to the data will be outlined in the relevant contract. The University of Oxford has a specialist insurance policy in place which would operate in the event of any participant suffering harm as a result of their involvement in the research.

### Qualitative interview methods

We will conduct one-to-one, semi-structured interviews with women and will recruit them in two ways. First, we intend to interview approximately 10–15 women who take part in the feasibility study. Women who consent to participate in the feasibility study will be asked whether they would be happy to be contacted by a member of the research team to take part in the interview study. Those who are happy to be contacted will be sent the CUTI interview study PIL with full details of the study, a reply slip and a freepost envelope (see Additional file 5 for the interview PIL). Second, we intend to interview approximately 10–15 women who are not participants of the study and have not been approached to take part in the study, but have experienced at least one UTI in the past 12 months. Interviewing this second group of women will afford us insights into barriers and facilitators to study recruitment for women who may be less amenable (compared with women who are taking part in the feasibility study) to trying non-antibiotic treatments for UTIs. Recruitment of this second group of women will take place concurrently with recruitment to the feasibility study; GP practices not involved with the feasibility study will conduct a search of their patient electronic records to identify potentially eligible participants – women aged 18 years and above who have had at least one UTI in the past 12 months. Where appropriate, they will then send relevant patients a participation invitation letter (on GP practice headed paper), a PIL, a reply slip and a freepost envelope.

Another means of recruitment will include advertising posters in GP practices and word of mouth; advertising posters will have tear-off slips at the bottom with contact details for the study team. If a potential participant is interested in taking part, they can directly contact the study team. An interview study PIL, reply slip and freepost envelope can then be sent to potentially eligible participants.

Written informed consent will be obtained by means of participant-dated signature and dated-signature of the person who presented and obtained the informed consent (see Additional file 6 for the interview ICF). A narrative, semi-structured interview guide [17] will be used to explore the participants’ experience of having a UTI, their thoughts on non-antibiotic treatments for UTIs and their thoughts on, or experience of, taking part in the feasibility study. The final number of interviews conducted will be determined by achieving data saturation [18]. As this is a small interview study with modest aims [19] to learn about the experience or perceived experience of participating in this trial, we anticipate that we will achieve saturation with a sample of between 20 and 30 participants. We expect that interviews will take no longer than 1 h, although the duration may vary from participant to participant.

### Data analysis

#### Objective 1 – Feasibility of undertaking this study in the primary care setting

Participants’ diary responses will be reviewed to determine study acceptability and the findings presented descriptively.

The data in the symptom diaries will be audited at the end of the study period to assess the quality and completeness of data capture. This will include review of the number of paper diaries, if any, that were provided to participants due to failure of the electronic diaries. The rate of recruitment of participants will also be assessed at the end of the study period, and the proportion of participants lost to follow-up from each arm will be reported.

Thematic analysis [20] will be used to develop themes from interview transcripts. The transcripts will be read several times for familiarity by the qualitative researcher, allowing immersion in the data. Codes will be generated based on the data and these will contribute to the generation of categories and analytic themes describing the meaning of the data. NVivo software will be used to aid in coding and organising the data. Data collection and analysis will take place concurrently, allowing an iterative evolution of the topic guide. This will also allow a deeper understanding of the emerging insights, which will inform further interviews.

#### Objective 2 – Preliminary assessment of the effect of cranberry on the number of antibiotic courses consumed and symptom burden

Exploratory calculations only will be attempted on the data from this feasibility study; we recognise that the study is not powered for estimates of effects. Nevertheless, we will pilot analysis of outcomes using a statistical package (e.g. Stata).

The proportion of participants consuming a course (or part thereof) of antibiotics in each group at 2 weeks and at the end of the follow-up period will be compared (group 2 compared with group 1, and group 3 compared with group 1) using a log binomial regression model. The treatment effects will be presented as relative risks. A Poisson regression model will be used to compare the number of antibiotic courses (or part thereof) consumed by participants in each group. Linear regression will be used to compare the symptom burden in each group, and time to event analysis will be used to analyse the time to resolution of symptoms rated ‘moderately bad’ or ‘worse’.

Participants’ notes will be reviewed to record any attendances to the GP practice, hospital or out-of-hours facilities within 28 days of the baseline visit that are believed to be related to the original UTI episode. The number of adverse events reported by participants (directly reported by participants and through review of diaries) and clinicians will be assessed, with the chi-squared test (or Fisher’s exact test if numbers are small) used to compare the proportion of AEs and SAEs in each arm of the study (comparison of total (S) AEs in each arm and proportion of participants with at least one (S) AE in each arm).

We will calculate the proportion of participants randomised to receive antibiotics alone who record consuming cranberry products in their completed symptom diary, with 95% confidence interval.

## Discussion

We are aware of an open-label RCT (EudraCT: 2018–001448-78) that is currently recruiting in Spain and assessing the non-inferiority of Cysticlean capsules (containing 240 mg PAC per capsule) compared with fosfomycin for the treatment of acute UTIs in women aged 18 to 64 years. To our knowledge, our study will be the first randomised clinical trial investigating the use of cranberry extract alone *and* as an adjunct to antibiotics in the management of acute UTIs in women. The feasibility study with embedded qualitative interviews will provide a platform for the conduct of a rigorous, adequately powered trial to determine whether or not cranberry is an effective treatment and/or adjunct to antibiotic treatment for acute uncomplicated UTIs.

We have not incorporated a placebo arm in this feasibility study for a number of reasons: this is not a mechanistic study; we intend to recruit small numbers of participants; and the study is not adequately powered for estimates of effects. However, arm 1 (usual practice – immediate antibiotics alone) serves as a control arm. This pragmatic approach is more closely reflective of day-to-day clinical practice [21]. In line with this practical approach, we have also not specified the antibiotic class, dose or duration that the healthcare practitioner should prescribe, recommending that they give ‘first-line antibiotics,’ but we recognise that this may vary according to participant characteristics and local guidance.

The findings will be disseminated through journal publication, blog publication and presentation at conferences. We will also provide a summary of the findings to participating practices.

## Trial status

Protocol version: 1.1, 10 June 2019.

The first site opened to recruitment on 1 July 2019. First participant recruited 11 July 2019. Approximate completion date 1 January 2020.

## Supplementary information

**Additional file 1.** Participant Information Leaflet (CUTI trial).

**Additional file 2.** Informed Consent Form (CUTI trial).

**Additional file 3.** Symptom diary.

**Additional file 4.** Standard Protocol Items: Recommendations for Interventional Trials (SPIRIT) 2013 Checklist: recommended items to address in a clinical trial protocol and related documents.

**Additional file 5.** Participant Information Leaflet (CUTI interview study).

**Additional file 6.** Informed Consent Form (CUTI interview study).

## Data Availability

Not applicable

## Abbreviations

AE: Adverse event
AMR: Antimicrobial resistance
CI: Chief Investigator
CRF: Case Report Form
DMC: Data Monitoring Committee
GP: General practitioner
ICF: Informed Consent Form
MSU: Mid-stream urine
PAC: Proanthocyanidins
PI: Principal Investigator
PIL: Participant Information Leaflet
RCT: Randomised clinical trial
REDCap: Research Data Capture
SAE: Serious adverse event
TSC: Trial Steering Committee
UTI: Urinary tract infection

## References

[1] Butler CC, Hawking MK, Quigley A, McNulty CA (2015). Incidence, severity, help seeking, and management of uncomplicated urinary tract infection: a population-based survey. Br J Gen Pract.

[2] Goossens H, Ferech M, Vander Stichele R, Elseviers M, Group EP (2005). Outpatient antibiotic use in Europe and association with resistance: a cross-national database study. Lancet.

[3] Alanis AJ (2005). Resistance to antibiotics: are we in the post-antibiotic era?. Arch Med Res.

[4] Gágyor I, Bleidorn J, Kochen MM, Schmiemann G, Wegscheider K, Hummers-Pradier E (2015). Ibuprofen versus fosfomycin for uncomplicated urinary tract infection in women: randomised controlled trial. BMJ.

[5] Kronenberg A, Bütikofer L, Odutayo A, Mühlemann K, da Costa BR, Battaglia M (2017). Symptomatic treatment of uncomplicated lower urinary tract infections in the ambulatory setting: randomised, double blind trial. BMJ.

[6] Vik I, Bollestad M, Grude N, Bærheim A, Damsgaard E, Neumark T (2018). Ibuprofen versus pivmecillinam for uncomplicated urinary tract infection in women—A double-blind, randomized non-inferiority trial. PLoS Med.

[7] Moore M, Trill J, Simpson C, Webley F, Radford M, Stanton L (2019). Uva-ursi extract and ibuprofen as alternative treatments for uncomplicated urinary tract infection in women (ATAFUTI): a factorial randomized trial.

[8] Howell AB, Reed JD, Krueger CG, Winterbottom R, Cunningham DG, Leahy M (2005). A-type cranberry proanthocyanidins and uropathogenic bacterial anti-adhesion activity. Phytochemistry..

[9] Jepson RG, Williams G, Craig JC. Cranberries for preventing urinary tract infections. Cochrane Database Syst Rev. 2012;10:CD001321. 10.1002/14651858.CD001321.pub5. 10.1002/14651858.CD001321.pub5 PMC7027998 23076891

[10] Papas P, Brusch C, Ceresia GJ (1966). Cranberry juice in the treatment of urinary tract infections. Southwest Med.

[11] Panchev P, Slavov C, Mladenov D, Georgiev M, Yanev K, Paskalev E (2012). A multicenter comparative observation on the effectiveness and the rapidness of the effect of Cystostop Rapid versus antibiotic therapy in patients with uncomplicated cystitis. Akush Ginekol (Sofiia).

[12] Little P, Moore M, Turner S, Rumsby K, Warner G, Lowes J (2010). Effectiveness of five different approaches in management of urinary tract infection: randomised controlled trial. BMJ..

[13] Trill J, Simpson C, Webley F, Radford M, Stanton L, Maishman T (2017). Uva-ursi extract and ibuprofen as alternative treatments of adult female urinary tract infection (ATAFUTI): study protocol for a randomised controlled trial. Trials..

[14] Howell AB, Botto H, Combescure C, Blanc-Potard A-B, Gausa L, Matsumoto T (2010). Dosage effect on uropathogenic *Escherichia coli* anti-adhesion activity in urine following consumption of cranberry powder standardized for proanthocyanidin content: a multicentric randomized double blind study. BMC Infect Dis.

[15] Butler CC, Francis N, Thomas-Jones E, Longo M, Wootton M, Llor C, et al. Point of care urine culture to inform appropriate antibiotic prescribing for uncomplicated urinary tract infection in primary care (POETIC): a randomised controlled trial of clinical and cost effectiveness. Awaiting Publ. 2017.

[16] Watson L, Little P, Moore M, Warner G, Williamson I (2001). Validation study of a diary for use in acute lower respiratory tract infection. Fam Pract.

[17] Smith JA, Harré R, Van Langenhove L. Rethinking methods in psychology. Thousand Oaks: Sage; 1995.

[18] Anselm S, Corbin J. Basics of qualitative research: techniques and procedures for developing grounded theory. Thousand Oaks: Sage; 1998.

[19] Charmaz K. Constructing grounded theory: a practical guide through qualitative analysis. Thousand Oaks: Sage; 2006.

[20] Braun V, Clarke V (2006). Using thematic analysis in psychology. Qual Res Psychol.

[21] Ford I, Norrie J (2016). Pragmatic trials. N Engl J Med.

