# Supplementary material for: Does cranberry extract reduce antibiotic use for symptoms of acute uncomplicated urinary tract infections (CUTI)? Protocol for a feasibility study
Source: Trials. 2019 Dec 23;20:767. doi: 10.1186/s13063-019-3860-z (PMC6929469; doi:10.1186/s13063-019-3860-z)
Supplement: Supplementary file 1 — Additional file 1. Participant Information Leaflet (CUTI trial). [file 13063_2019_3860_MOESM1_ESM.docx]

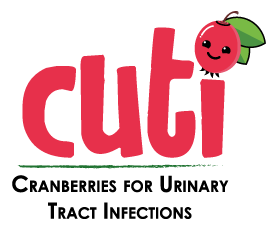


**PARTICIPANT INFORMATION LEAFLET (CUTI trial)**

You are being asked to take part in a research study. Before you decide if you want to participate or not, it is important for you to understand why the research is being done and what it will involve. This leaflet aims to tell you about the purpose of this study and what will happen to you if you decide to take part.

Please ask us if there is anything that is not clear or if you would like more information.

**CUTI Study Office:**

Nuffield Department of Primary Care Health Sciences

University of Oxford

Radcliffe Observatory Quarter

Oxford

OX2 6GG

Phone: 01865 289067

[
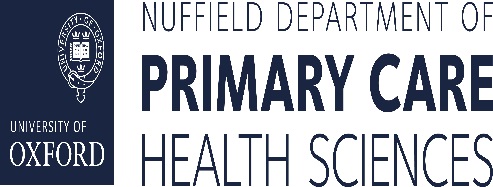
](https://www.google.co.uk/url?sa=i&source=images&cd=&ved=2ahUKEwi0mumojMzcAhVR1xoKHRGQD2IQjRx6BAgBEAU&url=https://www.phc.ox.ac.uk/intranet/communications-engagement/comms/brandguidelines&psig=AOvVaw25IKRdoU5fZKRI-OJ-Rdxv&ust=1533221189259821) [
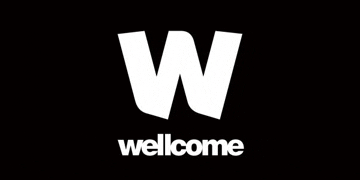
](https://www.google.co.uk/url?sa=i&source=images&cd=&ved=2ahUKEwiuxNPikczcAhWwyYUKHaUYAkAQjRx6BAgBEAU&url=https://jobs.newscientist.com/en-gb/employer/10006940/wellcome-trust/&psig=AOvVaw0mDDZCSW5l1hKxQkGqkRAC&ust=1533222668102409) [
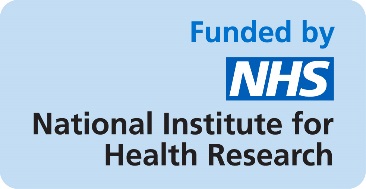
](http://ghrgst.nihr.ac.uk/about-us/)

**1. What is the purpose of the study?**

Urinary tract infections (UTIs) are the commonest bacterial infections affecting women, and are usually treated with antibiotics. Because of frequent and sometimes inappropriate use of antibiotics, many bacteria have adapted so that they are no longer killed by antibiotics (antibiotic resistance). As a result, there has been increasing interest in using non-antibiotic treatments, such as cranberry extract. The sugars in cranberries are believed to prevent bacteria from sticking to the wall of the bladder, thus reducing the ability of bacteria to cause a UTI. Cranberries might also make it easier for antibiotics to surround and kill bacteria.

Women with a suspected UTI are being invited to take part in this study and are being randomly assigned to one of three groups:

1) Group 1 - Usual treatment with antibiotics.

2) Group 2 - Treatment with antibiotics AND cranberry capsules.

3) Group 3 - Initial treatment with cranberry capsules. Women in this group will also be given an antibiotic prescription that they can take to the chemist at a later date if they don’t get better with cranberry capsules alone (‘back-up antibiotics’).

The cranberry capsules have been supplied by an Italian company called Indena S.p.A. Each capsule contains 60 mg of cranberry extract and 18 mg of proanthocyanidins (‘PAC’ for short – this is the active ingredient). We are not aware of any serious or common side effects that are associated with these capsules.

The main aim of this small-scale study is to test whether the study design described works and is acceptable to participants (called a ‘feasibility’ study). This will help in the planning of a subsequent, similar, but much larger study. By comparing the information from different treatment groups on a larger scale, we will be able to tell whether cranberries actually treat UTIs and help reduce the need for antibiotics. This study is being led by Dr Kome Gbinigie and forms part of her doctoral research.

**2. Why have I been invited?**

You have contacted a primary care provider with symptoms that suggest you might have a urinary tract infection.

**3. Do I have to take part?**

No. You are free to decide whether or not to take part. If you decide to take part you are still free to withdraw from the study at any time, without giving a reason. A decision not to take part or to withdraw will not affect the standard of care you receive from the healthcare team.

**4. What will happen to me if I agree to take part?**

- The clinician/healthcare provider/researcher will check if you are eligible to take part. This **is likely to** involve screening your medical records for relevant information and you will be asked to sign a consent form.
- You will then be randomly assigned (that is, like tossing a coin) to one of three ways of treating your UTI. The random assignment will be done electronically by your clinician/healthcare provider/researcher, by clicking a button. The three different ways are:

*Group 1*

An antibiotic prescription (usual practice)

*Group 2*

An antibiotic prescription AND a course of cranberry capsules to take for up to 7 days.

*Group 3*

An immediate course of cranberry capsules to take for up to 7 days AND a *delayed* prescription for antibiotics. You will be advised to take the antibiotics if your symptoms get worse, or don’t get better, within 3-5 days of taking cranberry capsules.

- You will be told which of the three groups you are in, and your clinician/healthcare provider/researcher will provide you with an antibiotic prescription and/or cranberry capsules. If you wish, you can also take pain relief/medicines to help your symptoms (e.g. paracetamol). However, if you are in group 1, we would ask you not to use any cranberry products/cranberry juice. If you are in groups 2 or 3, you will also be provided with a freepost envelope so that you can return any unused cranberry capsules back to the study team. You will be given a participant information pack, which will include a guide on how to complete the electronic symptom diary.
- You will be asked to provide a urine sample (if you have not already provided a sample as part of your routine care). This will be sent to a laboratory for analysis to see whether any bugs grow.
- You will be emailed a link to an electronic questionnaire (a symptom diary). There are three sections to the symptom diary. The first section should be completed on the same day that you agreed to participate in the study (i.e. the day that you saw a healthcare professional/clinician/researcher), and will ask you some background questions (such as whether you have had a urinary tract infection in the past).
- You will need to keep the electronic link to the questionnaire safe and click on it every day for up to 2 weeks (depending on how long you have symptoms for). This part of the questionnaire will allow you to rate how bad your symptoms are and to enter the medications that you are taking.
- Two weeks from now, you will be asked to complete the third section of the electronic symptom diary. In this section you will be asked further questions, including how easy/difficult you found it to use and complete the electronic symptom diary.
- You will also receive an email +/- telephone call two weeks from now from a member of the study team, principally to ensure that you managed to complete the electronic symptom diary.
- Please note that if you are in group 2 or group 3 (i.e. a group that receives cranberry capsules), you will not receive cranberry capsules beyond your period of involvement with the study.
- When you are consented to take part in this study, we will also ask you whether you would be happy to be contacted to take part in an interview to discuss your thoughts on taking part in this study as well as your thoughts on the management of UTIs more generally. There is no obligation for you to take part in the interview study; declining to take part in the interview study will not affect your participation in this study or the level of care provided to you by your healthcare provider. If you decide to also take part in the interview study, you will be provided with more detailed information about what to expect and separate informed consent will be taken. The interview will be arranged at a time to suit you. We anticipate that the interview should take no longer than an hour and can be carried out in person, in your own home or other preferred place, or by telephone, whichever you prefer. The interview will take place between 1 and 6 months from now.
- After one month, your medical notes will be reviewed by a member of the study team (possibly your GP) to find out the results of your urine culture that was sent to the laboratory and also to see whether there have been any side effects/problems related to your participation in the study.

**5. What will happen to my urine sample?**

Your urine sample will be sent to a laboratory for analysis as per the usual practice of your GP surgery/primary care provider. The urine sample will be destroyed in accordance with usual laboratory procedures.

At a later stage, a member of the study team who is authorised to do so will access your medical notes to determine whether or not your urine grew a bug. This may be a GP at your surgery.

**6. What are the possible disadvantages and risks of taking part?**

There are no known serious or common side effects associated with taking cranberry capsules.

If you are randomly assigned to group three, you will not receive immediate antibiotics. This may mean that you are at higher risk of developing an upper UTI. ***Irrespective of the group to which you are assigned, if you develop any symptoms that suggest that you have an upper UTI (pain in your back, pain in your side, fever and/or vomiting), you must seek urgent medical attention.*** It is likely that you will require a 7 day course of antibiotics. If you decide to take part in this study, this information will also be in your participant information pack.

**7. What are the possible benefits of taking part?**

If you complete the electronic symptom diary, you will receive a £10 voucher as a thank you for taking part, after you have finished taking part in the study. If you also take part in the interview, you will receive a further £10 voucher after the interview has been completed.

Taking part in this study will help us to design a similar study to this but on a much larger scale (with more participants and in different parts of the country). The results of the larger study may help us to safely reduce antibiotic use in women with simple urinary tract infections.

**8. Will my taking part in this study be kept confidential?**

Yes. The study will comply with the General Data Protection Regulation (GDPR) and Data Protection Act 2018 and all information collected about you during the research will be kept strictly confidential. Only the relevant sections of your medical notes will be reviewed. Responsible members of the University of Oxford may be given access to data for monitoring and/or audit of the study to ensure that the research is complying with applicable regulations. You will be assigned a participant ID number and identified by this on study documents and electronic databases.

**9. What will happen to my data?**

Data protection regulation requires that we state the legal basis for processing information about you. In the case of research, this is ‘a task in the public interest.’ The University of Oxford is the data controller and is responsible for looking after your information and using it properly.

We will be using information from your medical records and will use the minimum personally-identifiable information possible. We will keep identifiable information about you for 6-12 months after the study has finished. We will store the de-identified research data and any research documents with personal information, such as consent forms, securely at the University of Oxford for 5 years after the end of the study. Data protection regulation provides you with control over your personal data and how it is used. When you agree to your information being used in research, however, some of those rights may be limited in order for the research to be reliable and accurate. Further information about your rights with respect to your personal data is available at: <http://www.admin.ox.ac.uk/councilsec/compliance/gdpr/individualrights/>]

Data which does not identify you will be stored by the study team and may be used in future research projects.

You can find out more about how we use your information by contacting the Chief Investigator using the details at the end of this sheet.

**10. What if I do not want to take part and/or do not want to carry on with the study once I have started?**

If you do not want to take part in the research you can withdraw at any time without affecting your care. Information collected up to your withdrawal will be kept. Withdrawing from the study will not affect the standard of care you receive from the healthcare team. If you would like to withdraw at any time we would ask you to let one of the research team know. If you lose the ability to confirm that you are happy to proceed with the study (i.e. if you lose the capacity to consent to ongoing participation), information collected up until this point will be kept.

**11. What if there is a problem?**

If you wish to complain about any aspect of the way in which you have been approached or treated, or how your information is handled during the course of this study, you should contact Dr Kome Gbinigie (Chief Investigator) on 01865 289067 or [cuti@phc.ox.ac.uk](mailto:cuti@phc.ox.ac.uk), or you may contact the University of Oxford Clinical Trials and Research Governance (CTRG) office on 01865 616480, or the head of CTRG, email [ctrg@admin.ox.ac.uk](mailto:ctrg@admin.ox.ac.uk). The University of Oxford, as Sponsor, has appropriate insurance in place in the unlikely event that you suffer any harm as a direct consequence of your participation in this study.

**12. What will happen to the results of the research study?**

We aim to publish the results so that scientists and doctors are aware of the findings. We will also provide your GP practice a copy of our findings for them to display. You will not be personally identifiable in any publication.

**13. Who is organizing and funding the research?**

This study is sponsored by the University of Oxford and the funding for this research comes from the NIHR School for Primary Care (SPCR) and the Wellcome Trust. Indena S.p.A (an Italian company) have supplied the cranberry capsules (Redicran capsules) for this study; they have no other involvement with the study.

**14. Who has reviewed this study?**

All research in the NHS is looked at by an independent group of people, called a Research Ethics Committee, to protect participants’ interests. This study has been reviewed and given favourable opinion by South Central Research Ethics Committee.

**15. Do you have any further questions or concerns?**

If you want to discuss the study please contact Dr Kome Gbinigie, Chief Investigator, by email (cuti@phc.ox.ac.uk) or telephone (01865 289067).

**Thank you for taking the time to read this information leaflet**
