# Supplementary material for: Does cranberry extract reduce antibiotic use for symptoms of acute uncomplicated urinary tract infections (CUTI)? Protocol for a feasibility study
Source: Trials. 2019 Dec 23;20:767. doi: 10.1186/s13063-019-3860-z (PMC6929469; doi:10.1186/s13063-019-3860-z)
Supplement: Supplementary file 2 — Additional file 2. Informed Consent Form (CUTI trial). [file 13063_2019_3860_MOESM2_ESM.docx]

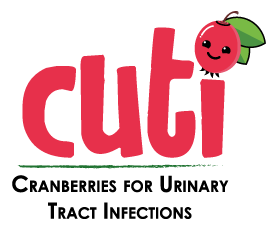
 **INFORMED CONSENT FORM – CUTI Trial**

**Chief Investigator:** Dr Kome Gbinigie. **Address:** Nuffield Department of Primary Care Health Sciences, Radcliffe Primary Care building, Radcliffe Observatory Quarter, Woodstock Road, Oxford, OX2 6GG. **Email:** cuti@phc.ox.ac.uk **Telephone:** ­­01865 289067

**Study Title:** CUTI trial **Participant** **ID number: ______________________**

If you are happy to take part in this research then please read each of the statements below and initial the boxes if you agree with them.

**Please initial each box**

1. I confirm that I have read and understood the information sheet version [number], dated [date month year] for the CUTI trial and have had the opportunity to ask questions and had these answered satisfactorily.

2. I understand that my participation is voluntary and that I am free to withdraw at any time, without giving any reason, and without my medical care or legal rights being affected.

3. I understand that the study team will have access to the results of the urine culture test performed for my UTI episode.

4. I understand that relevant sections of my medical notes will be reviewed after 28 days.

5. I understand that relevant sections of my medical notes and data collected during the study may be looked at by individuals from the University of Oxford or regulatory authorities, where it is relevant to my taking part in this research. I give permission for these individuals to have access to my records.

6. I understand that the information that I have given to researchers will be transferred to Oxford University and stored securely. I give permission for the research team to use de-identified data collected as part of this study for future research.

7. I give permission for de-identified quotes from the patient symptom diary to be included in reports of the findings from the research.

8. By signing this consent form, I agree to participate in the CUTI trial and follow all trial procedures.

**ADDITIONAL STUDY –** Please initial either the yes (Y) or no (N) box:

**N**

**Y**

8. I am happy to be contacted regarding an interview study (CUTI Interview study) aiming to find out what patients think about the management of UTIs, their experience of having a UTI and their experience of the CUTI trial.

**Participant name:…………………………………………………….. Name of person taking consent:………………………………………………………**

**Date:……………………………………………………………………….. Date:……………………………………………………………………………………………….**

**Signature:……………………………………………………………….. Signature:……………………………………………………………………………………….**

***When completed, one copy should be given to the participant, one copy should stay at the study site and one copy should go in the participant’s medical notes***

[
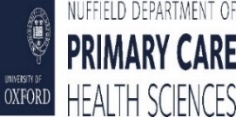
](https://www.google.co.uk/url?sa=i&source=images&cd=&ved=2ahUKEwi0mumojMzcAhVR1xoKHRGQD2IQjRx6BAgBEAU&url=https://www.phc.ox.ac.uk/intranet/communications-engagement/comms/brandguidelines&psig=AOvVaw25IKRdoU5fZKRI-OJ-Rdxv&ust=1533221189259821) [
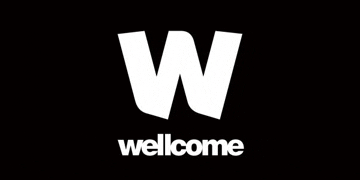
](https://www.google.co.uk/url?sa=i&source=images&cd=&ved=2ahUKEwiuxNPikczcAhWwyYUKHaUYAkAQjRx6BAgBEAU&url=https://jobs.newscientist.com/en-gb/employer/10006940/wellcome-trust/&psig=AOvVaw0mDDZCSW5l1hKxQkGqkRAC&ust=1533222668102409) [
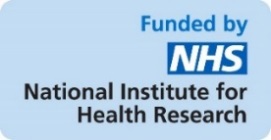
](http://ghrgst.nihr.ac.uk/about-us/)
