# Supplementary material for: Does cranberry extract reduce antibiotic use for symptoms of acute uncomplicated urinary tract infections (CUTI)? Protocol for a feasibility study
Source: Trials. 2019 Dec 23;20:767. doi: 10.1186/s13063-019-3860-z (PMC6929469; doi:10.1186/s13063-019-3860-z)
Supplement: Supplementary file 3 — Additional file 3. Symptom diary. [file 13063_2019_3860_MOESM3_ESM.docx]

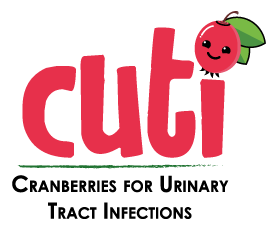


**Patient ID:**

**Age:** _______ Years

**Date of Registration:** DD/MM/YYYY

**Site ID:**

**PLEASE COMPLETE THIS TWO WEEK DIARY. IT IS DIVIDED INTO THREE SECTIONS AS EXPLAINED BELOW:**

**Section 1:** Please complete this section **on the day** you saw your health practitioner.

**Section 2:** Please complete this section **every day over the next two weeks or until symptoms subside and no further treatments are being used.**

**Section 3:** Please complete this section at the **end of the two weeks.**

[
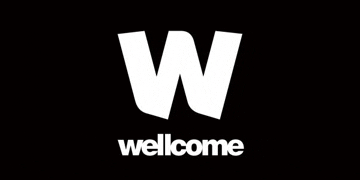
](https://www.google.co.uk/url?sa=i&source=images&cd=&ved=2ahUKEwiuxNPikczcAhWwyYUKHaUYAkAQjRx6BAgBEAU&url=https://jobs.newscientist.com/en-gb/employer/10006940/wellcome-trust/&psig=AOvVaw0mDDZCSW5l1hKxQkGqkRAC&ust=1533222668102409) [
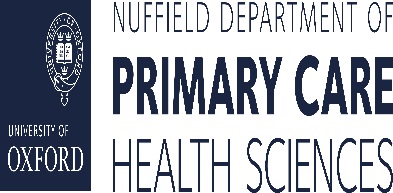
](https://www.google.co.uk/url?sa=i&source=images&cd=&ved=2ahUKEwi0mumojMzcAhVR1xoKHRGQD2IQjRx6BAgBEAU&url=https://www.phc.ox.ac.uk/intranet/communications-engagement/comms/brandguidelines&psig=AOvVaw25IKRdoU5fZKRI-OJ-Rdxv&ust=1533221189259821) [
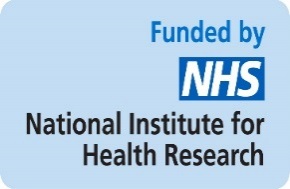
](http://ghrgst.nihr.ac.uk/about-us/)

**SECTION 1: ABOUT YOU.** Please complete this section on the day you saw your doctor, nurse, healthcare practitioner or researcher.

PART A – Age in years

PART B – History of Urine Infections

1) Have you ever had a urine infection diagnosed by a doctor or nurse at any point in the past (not including the current infection)?

Yes

No

Do not know

*If you answered* ***no*** *or* ***do not know*** *then go to Part C*

2) Have you had a urine infection in the past year (not including the current infection)?

Yes

No

3) How many times have you been treated for a urine infection in the past year (not including the current infection)?

Zero

One

Two

Three or more

Unable to remember

How many months has it been since your last urine infection (not including the current infection)?

Months

Do not remember - *tick this box if you cannot remember how many months it has been.*

4) How was your last urine infection treated (not including the current infection)? *Please tick all relevant boxes*

Antibiotics -> Name of antibiotic(s) (if known)

Other -> Please specify

No treatment

Do not remember

PART C – Additional Information

1) When you contacted your GP/healthcare practitioner for your current urine infection were you expecting to receive any of the following? *Please tick one box for each option*

Were you expecting antibiotics?

Yes

No

Unsure

Were you expecting tests/investigations?

Yes

No

Unsure

Were you expecting advice?

Yes

No

Unsure

Were you expecting anything else?

Yes -> Please specify

No

Unsure

2) Before you went to see your doctor/healthcare practitioner did you try and manage your urine infection with any of the following? *Please tick all that apply.*

Cranberry juice/cranberry products

Other fruit juice

Bicarbonate solution

Sodium/Potassium citrate (cystitis sachets)

Uvacin/Uva ursi (bearberry)

Pain relief (e.g. paracetamol/Ibuprofen)

Other If other, please specify

None

3) Before contacting your GP practice/healthcare provider today, for how many days have you felt that you had symptoms of a urinary infection? *Do not include today.*

0 days (that is, your symptoms started today)

1 day

2 days

3 days

More than 3 days

4) Do you believe that cranberry juice/cranberry products might help your symptoms?

Yes

No

Unsure

5) Do you believe that your symptoms can get better without antibiotics?

Yes

No

Unsure

**SECTION 2: TWO WEEK DIARY OF URINE INFECTION SYMPTOMS/PROBLEMS/TREATMENTS**

Please fill in the diary on the next page and record your symptoms and any treatments (study medication, antibiotics, other medications or products) used. Please start **THIS EVENING** (the evening of the day on which you saw your GP/nurse/healthcare practitioner/researcher) and continue to fill this in each evening for 2 weeks or until all symptoms have completely gone and no treatments are being taken.

If all of your symptoms are rated as 0 for two days in a row you no longer need to complete the symptom scoring chart; please enter the date in the relevant box. However, please continue to record the medication that you have taken and complete Section 3, 2 weeks after you first saw the doctor/nurse.

For each symptom/problem, rate how bad it has been using the scale on the following page. The rating that you give should reflect how you have felt over the past 24 hours.

**WEEK 1 SYMPTOMS**

For each symptom/problem, please rate how bad it has been using the scale below. If you have no symptoms or problems, please enter 0 (to indicate normal/not affected.) The shaded column is completed as an example – please fill in your own numbers:

0 – Normal/not affected

1-9 – Very little problem

10-19 – Slight problem

20-29 – Moderately bad

30-39 – Bad

40-49 – Very Bad

50 – As bad as it could be

| DAY | E.g. | 1  (The day you were seen by your GP/nurse/  Healthcare practitioner) | 2 | 3 | 4 | 5 | 6 | 7 |
| --- | --- | --- | --- | --- | --- | --- | --- | --- |
| DAY OF WEEK | Mon |  |  |  |  |  |  |  |
| SYMPTOM/PROBLEM |  |  |  |  |  |  |  |  |
| Fever | 0 |  |  |  |  |  |  |  |
| Pain in the side of tummy | 35 |  |  |  |  |  |  |  |
| Blood in urine | 0 |  |  |  |  |  |  |  |
| Smelly urine | 20 |  |  |  |  |  |  |  |
| Burning or pain when passing urine | 46 |  |  |  |  |  |  |  |
| Having to pass urine in a hurry (urgency) | 15 |  |  |  |  |  |  |  |
| Passing urine *more often than* *usual* during the day (Day time frequency) | 18 |  |  |  |  |  |  |  |
| Getting up at night *more than usual* to pass urine (Night time frequency) | 12 |  |  |  |  |  |  |  |
| Lower tummy pain (When not passing urine) | 0 |  |  |  |  |  |  |  |
| Restricted activities | 0 |  |  |  |  |  |  |  |
| Feeling generally unwell | 6 |  |  |  |  |  |  |  |

**WEEK 1 TREATMENTS**

Please also enter any medication that you are taking for your urine infection each day. Please indicate:

- The **medications/treatments** you are taking for your urine infection**.** This includes prescribed medications (such as antibiotics and cranberry capsules) and any medications that you have purchased over the counter. If a medication that you are taking is not listed in the table below, please add at the bottom in one of the empty rows.
- The **number of times** that you have taken the **medication/treatment each day** (Please note that you only need to record the number of times in the day that you have taken the medication, not the number of capsules/tablets/sachets taken each day).
- The shaded row is completed as an example – please fill in your own answers.

| **Medication** | **Day 1** | | **Day 2** | | **Day 3** | | | **Day 4** | | | | **Day 5** | | | | | **Day 6** | | | | **Day 7** | | |
| --- | --- | --- | --- | --- | --- | --- | --- | --- | --- | --- | --- | --- | --- | --- | --- | --- | --- | --- | --- | --- | --- | --- | --- |
|  | Taken today? (Y/N) | How many times? | Taken today? (Y/N) | How many times? | Taken today? (Y/N) | How many times? | | Taken today? (Y/N) | | How many times? | | Taken today? (Y/N) | | | How many times? | | Taken today? (Y/N) | | How many times? | | Taken today? (Y/N) | | How many times? |
| e.g. Paracetamol | Y | 4 | Y | 4 | N | |  | | Y | | 2 | | Y | 3 | | N | |  | | N | |  | |
| Nitrofurantoin |  |  |  |  |  | |  | |  | |  | |  |  | |  | |  | |  | |  | |
| Trimethoprim |  |  |  |  |  | |  | |  | |  | |  |  | |  | |  | |  | |  | |
| Amoxicillin |  |  |  |  |  | |  | |  | |  | |  |  | |  | |  | |  | |  | |
| Pivmecillinam |  |  |  |  |  | |  | |  | |  | |  |  | |  | |  | |  | |  | |
| Fosfomycin |  |  |  |  |  | |  | |  | |  | |  |  | |  | |  | |  | |  | |
| Co-amoxiclav |  |  |  |  |  | |  | |  | |  | |  |  | |  | |  | |  | |  | |
| Ciprofloxacin |  |  |  |  |  | |  | |  | |  | |  |  | |  | |  | |  | |  | |
| Cranberry capsules |  |  |  |  |  | |  | |  | |  | |  |  | |  | |  | |  | |  | |
| Paracetamol |  |  |  |  |  | |  | |  | |  | |  |  | |  | |  | |  | |  | |
| Ibuprofen |  |  |  |  |  | |  | |  | |  | |  |  | |  | |  | |  | |  | |
| Cystitis sachets |  |  |  |  |  | |  | |  | |  | |  |  | |  | |  | |  | |  | |
|  |  |  |  |  |  | |  | |  | |  | |  |  | |  | |  | |  | |  | |
|  |  |  |  |  |  | |  | |  | |  | |  |  | |  | |  | |  | |  | |
|  |  |  |  |  |  | |  | |  | |  | |  |  | |  | |  | |  | |  | |

**WEEK 2 SYMPTOMS**

For each symptom/problem, please rate how bad it has been using the scale below. If you have no symptoms or problems, please enter 0 (to indicate normal/not affected.) The shaded column is completed as an example – please fill in your own numbers:

0 – Normal/not affected

1-9 – Very little problem

10-19 – Slight problem

20-29 – Moderately bad

30-39 – Bad

40-49 – Very Bad

50 – As bad as it could be

| DAY | E.g. | 8 | 9 | 10 | 11 | 12 | 13 | 14 |
| --- | --- | --- | --- | --- | --- | --- | --- | --- |
| DAY OF WEEK | Mon |  |  |  |  |  |  |  |
| SYMPTOM/PROBLEM |  |  |  |  |  |  |  |  |
| Fever | 0 |  |  |  |  |  |  |  |
| Pain in the side of tummy | 35 |  |  |  |  |  |  |  |
| Blood in urine | 0 |  |  |  |  |  |  |  |
| Smelly urine | 20 |  |  |  |  |  |  |  |
| Burning or pain when passing urine | 46 |  |  |  |  |  |  |  |
| Having to pass urine in a hurry (urgency) | 15 |  |  |  |  |  |  |  |
| Passing urine *more often than* *usual* during the day (Day time frequency) | 18 |  |  |  |  |  |  |  |
| Getting up at night *more than usual* to pass urine (Night time frequency) | 12 |  |  |  |  |  |  |  |
| Lower tummy pain (When not passing urine) | 0 |  |  |  |  |  |  |  |
| Restricted activities | 0 |  |  |  |  |  |  |  |
| Feeling generally unwell | 6 |  |  |  |  |  |  |  |

**WEEK 2 TREATMENTS**

Please also enter any treatments that you are taking for your urine infection each day. Please indicate:

- The **medications/treatments** you are taking for your urine infection**.** This includes prescribed medications (such as antibiotics and cranberry capsules) and any medications that you have purchased over the counter. If a medication that you are taking is not listed in the table below, please add at the bottom in one of the empty rows.
- The **number of times** that you have taken the **medication/treatment each day** (Please note that you only need to record the number of times in the day that you have taken the medication, not the number of capsules/tablets/sachets taken each day).
- The shaded row is completed as an example – please fill in your own answers.

| **Medication** | **Day 8** | | **Day 9** | | **Day 10** | | | **Day 11** | | | | **Day 12** | | | | | **Day 13** | | | | **Day 14** | | |
| --- | --- | --- | --- | --- | --- | --- | --- | --- | --- | --- | --- | --- | --- | --- | --- | --- | --- | --- | --- | --- | --- | --- | --- |
|  | Taken today? (Y/N) | How many times? | Taken today? (Y/N) | How many times? | Taken today? (Y/N) | How many times? | | Taken today? (Y/N) | | How many times? | | Taken today? (Y/N) | | | How many times? | | Taken today? (Y/N) | | How many times? | | Taken today? (Y/N) | | How many times? |
| e.g. Paracetamol | Y | 3 | N |  | N | |  | | N | |  | | N |  | | N | |  | | N | |  | |
| Nitrofurantoin |  |  |  |  |  | |  | |  | |  | |  |  | |  | |  | |  | |  | |
| Trimethoprim |  |  |  |  |  | |  | |  | |  | |  |  | |  | |  | |  | |  | |
| Amoxicillin |  |  |  |  |  | |  | |  | |  | |  |  | |  | |  | |  | |  | |
| Pivmecillinam |  |  |  |  |  | |  | |  | |  | |  |  | |  | |  | |  | |  | |
| Fosfomycin |  |  |  |  |  | |  | |  | |  | |  |  | |  | |  | |  | |  | |
| Co-amoxiclav |  |  |  |  |  | |  | |  | |  | |  |  | |  | |  | |  | |  | |
| Ciprofloxacin |  |  |  |  |  | |  | |  | |  | |  |  | |  | |  | |  | |  | |
| Cranberry capsules |  |  |  |  |  | |  | |  | |  | |  |  | |  | |  | |  | |  | |
| Paracetamol |  |  |  |  |  | |  | |  | |  | |  |  | |  | |  | |  | |  | |
| Ibuprofen |  |  |  |  |  | |  | |  | |  | |  |  | |  | |  | |  | |  | |
| Cystitis sachets |  |  |  |  |  | |  | |  | |  | |  |  | |  | |  | |  | |  | |
|  |  |  |  |  |  | |  | |  | |  | |  |  | |  | |  | |  | |  | |
|  |  |  |  |  |  | |  | |  | |  | |  |  | |  | |  | |  | |  | |
|  |  |  |  |  |  | |  | |  | |  | |  |  | |  | |  | |  | |  | |

Did your symptoms go away completely at some point over the past 2 weeks?

Yes

No

If you answered **no,** please skip to section 3.

What was the date that you felt completely recovered?

/ /

d d m m y y y y

**SECTION 3:** Please complete this section two weeks (14 days) after you saw your doctor/nurse/health care practitioner

**Since you saw your doctor/nurse/healthcare practitioner:**

1) Have you consulted a healthcare professional from your **general practice** or **out of hours’ provider** about your urine infection? *(Do not include the original visit)*

Yes

No

If you answered **YES** then who did you see? Please enter the number of times you saw them in the box. If you did not see the person in question then please enter 0.

GP at surgery

Nurse at surgery

GP at home

Out of hours’ doctor

Other If other, please specify

2) Have you consulted with a healthcare professional in an **accident and emergency** department about your urine infection?

Yes

No

If you answered **YES**, then how many times?

3) Have you been seen by a specialist (not including an admission to hospital) about your urine infection? This might occur if your GP or another healthcare professional had referred you for an urgent opinion about your urine infection, but you were not admitted to hospital.

Yes

No

If you answered **YES**, then how many times?

4) Have you been admitted to hospital for a problem related to your urine infection?

Yes

No

If you answered **YES**, then how many nights did you spend in hospital?

5) Have you used any of the following to try and manage your urine infection since your original consultation? *Not including study medication that you may or may not have received.* *Please tick all that apply*

Cranberry juice/cranberry products

Other fruit juice

Bicarbonate solution

Sodium/Potassium citrate (cystitis sachets)

Uvacin/Uva ursi (bearberry)

Pain relief (e.g. paracetamol/ibuprofen)

Other If other, please specify

None

6) Did you take any time off paid work/your usual activities because of your urine infection?

Yes

No

If you answered **YES**, then how many days? *Please round to the nearest day.*

7) **If you were prescribed cranberry capsules**, did you experience any side effects or problems related to taking the capsules? *If you were not prescribed cranberry capsules, please skip to question 8.*

Yes

No

If you answered **YES**, please explain the problems that you experienced in the box below.

8) Did you have any problems completing this 2 week diary?

No problems

Some problems

Lots of problems

If you experienced **some problems** or **lots of problems** in completing this diary, please explain the difficulty that you experienced in the box below.

9) Did you have any problems using a scale of 0-50 to rate your symptoms?

No problems

Some problems

Lots of problems

If you experienced **some problems** or **lots of problems** using the scale of 0-50 to rate your symptoms, please explain the difficulty that you experienced in the box below.

**That is the end of the questions!**

Thank you very much for completing the questions. You have made a valuable contribution to this important medical research. The information that you have provided will remain confidential.

**Please add in any comments you have about this study or urine infections.**

If you have any problems or queries, please contact:

Dr Kome Gbinigie,

­­­­­CUTI Trial Manager

Radcliffe Primary Care Building, Radcliffe Observatory Quarter

Woodstock Road

Oxford

OX2 6GG

Phone: 01865 289067
