# Supplementary material for: Does cranberry extract reduce antibiotic use for symptoms of acute uncomplicated urinary tract infections (CUTI)? Protocol for a feasibility study
Source: Trials. 2019 Dec 23;20:767. doi: 10.1186/s13063-019-3860-z (PMC6929469; doi:10.1186/s13063-019-3860-z)
Supplement: Supplementary file 5 — Additional file 5. Participant Information Leaflet (CUTI interview study). [file 13063_2019_3860_MOESM5_ESM.docx]

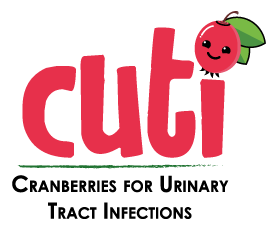


**PARTICIPANT INFORMATION LEAFLET (CUTI Interview study)**

You are being asked to take part in an interview study. Before you decide if you want to take part or not, it is important for you to understand why the research is being done and what it will involve. This leaflet aims to tell you about the purpose of this study and what will happen to you if you decide to take part.

Please ask us if there is anything that is not clear or if you would like more information.

**CUTI Study Office:**

Nuffield Department of Primary Care Health Sciences

University of Oxford

Radcliffe Observatory Quarter

Oxford

OX2 6GG

**Phone:** 01865 289067

[
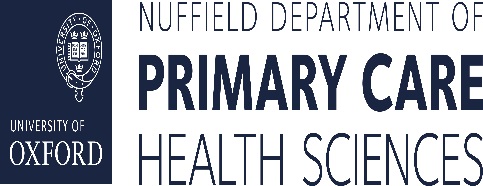
](https://www.google.co.uk/url?sa=i&source=images&cd=&ved=2ahUKEwi0mumojMzcAhVR1xoKHRGQD2IQjRx6BAgBEAU&url=https://www.phc.ox.ac.uk/intranet/communications-engagement/comms/brandguidelines&psig=AOvVaw25IKRdoU5fZKRI-OJ-Rdxv&ust=1533221189259821) [
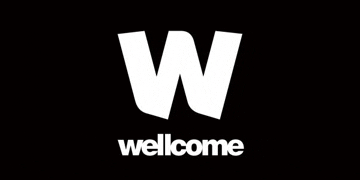
](https://www.google.co.uk/url?sa=i&source=images&cd=&ved=2ahUKEwiuxNPikczcAhWwyYUKHaUYAkAQjRx6BAgBEAU&url=https://jobs.newscientist.com/en-gb/employer/10006940/wellcome-trust/&psig=AOvVaw0mDDZCSW5l1hKxQkGqkRAC&ust=1533222668102409) [
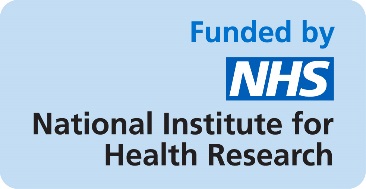
](http://ghrgst.nihr.ac.uk/about-us/)

**1. What is the purpose of the study?**

We are currently running a trial (called the ‘CUTI’ trial) in which women are being divided into three treatment groups to test whether cranberry capsules can help treat urinary tract infections (UTIs). Group 1 receive antibiotics to treat their UTI, group 2 receive antibiotics *and* cranberry capsules, and group 3 receive cranberry capsules with a back-up antibiotic prescription in case they don’t get better with cranberry capsules alone. The main aim of this trial is to test whether the way we do this research works and is acceptable to the women who take part (called a ‘feasibility trial’). This will help us to plan a similar but bigger trial. By comparing the information from different treatment groups in the bigger trial, we will be able to tell whether cranberries actually treat UTIs, reducing the need for antibiotics.

In order to help us understand better how to run the bigger trial successfully, in addition to comparing measurable data from the feasibility trial (e.g. the *number* of people in each group who required antibiotics), we also need to explore data that cannot be measured (e.g. how women *feel* about taking part in the feasibility trial). The best way to do this is through interviews (called ‘qualitative research’).

We intend to interview some women who take part in the feasibility trial, as well as some women who do not participate in the trial but have experience of UTIs. We want to explore women’s experience of having UTIs, what influences the way they seek help for UTIs, their attitudes towards self-care/alternative treatments, their experience (if applicable) of participating in the CUTI trial and what might influence somebody to want, or not want, to take part in the CUTI trial.

This information, combined with measurable data that we obtain from the feasibility trial, will provide deep insights into why our feasibility trial does or doesn’t work, and will help us to plan a successful bigger trial. This study is being led by Dr Kome Gbinigie and forms part of her doctoral research.

**2. Why have I been invited?**

We are inviting two groups of women. You are being invited because either:

1) You are also taking part in the CUTI trial.

2) You are not taking part in the CUTI, but you have experienced at least one UTI in the past year.

We would like to interview you to find out your experience of having had a UTI, what influences the way you seek help for UTIs, your attitude towards self-care/alternative treatments (e.g. cranberry capsules), your experience (if applicable) of participating in the CUTI trial and also what might influence you to want, or not want, to take part in the CUTI trial.

**3. Do I have to take part?**

No. You are free to decide whether or not to take part. If you decide to take part you are still free to withdraw from the study at any time, without giving a reason. A decision not to take part or to withdraw will not affect the standard of care you receive from the healthcare team.

**4. What will happen to me if I agree to take part?**

The interview will be arranged at a time to suit you. We anticipate that the interview should take no longer than an hour and can be carried out in person, in your own home or other preferred place, or by telephone, whichever you prefer. The interview will be audio recorded with your consent.

Before starting the interview you will be asked to sign a form (called a ‘consent’ form) to confirm that you fully understand what taking part in the study will involve and that your questions about the research have been answered. If you decide to be interviewed by telephone, we will ask you to sign a consent form and post it back to the research team before the telephone interview takes place. You will be provided with a freepost envelope.

If you are interested in taking part please contact the research team by returning your reply slip or by contacting the research team using the details provided in Section 14 below. The research team will be able to answer any questions you have and make sure you understand the study information. You will be given as long as you need to decide whether or not to take part.

**5. What are the possible disadvantages and risks of taking part?**

We do not anticipate that the interview will cause you emotional distress. If at any stage you do not wish to answer a question, that’s fine. Just let the researcher know. If you are feeling distressed during the interview, you can take a break, continue on another day, or stop the interview altogether. You may also wish to speak to your GP for support.

**6. What are the possible benefits of taking part?**

After you have completed the interview, you will receive a £10 voucher as a thank you for taking part in this study.

Taking part in this study will help us to design a large study to test the use of cranberry capsules in treating UTIs in women. The results of the larger study may help us to safely reduce antibiotic use in women with simple UTIs.

**7. Will my taking part in this study be kept confidential?**

With your permission the interview will be audio-recorded to make an accurate record of what is said and then the recording will be stored on a secure computer on University of Oxford premises. The recording will be sent securely to an independent transcription company who will type up the recording. This company will hold a confidential agreement with the University of Oxford. This written record will not include the names or other defining details that can identify you, to ensure confidentiality. Once the recording has been transcribed, the recording will be stored securely on University of Oxford premises until the end of the study and then deleted. De-identified transcripts will be stored at the University of Oxford and may be accessed by researchers for future research studies. Other study documents will be stored for 5 years following the end of the study and then destroyed. Responsible members of the University of Oxford may be given access to data for monitoring and/or audit of the study to ensure that the research is complying with applicable regulations. The findings from this study will be used in research reports but no names will be included in the report so any quotes from the interview will be de-identified. The only time that your interview would not remain confidential is if it were possible that you or someone else were at risk, but this would be discussed with you first. You will be assigned a participant ID number and identified by this on study documents and electronic databases.

**8. What will happen to my data?**

Data protection regulation requires that we state the legal basis for processing information about you. In the case of research, this is ‘a task in the public interest.’ The University of Oxford is the data controller and is responsible for looking after your information and using it properly.

We will be using information from your medical records and will use the minimum personally-identifiable information possible. We will keep identifiable information about you for 6-12 months after the study has finished. We will store the de-identified research data and any research documents with personal information, such as consent forms, securely at the University of Oxford for 5 years after the end of the study. Data protection regulation provides you with control over your personal data and how it is used. When you agree to your information being used in research, however, some of those rights may be limited in order for the research to be reliable and accurate. Further information about your rights with respect to your personal data is available at: <http://www.admin.ox.ac.uk/councilsec/compliance/gdpr/individualrights/>]

Data which does not identify you will be stored by the study team and may be used in future research projects.

You can find out more about how we use your information by contacting the Chief Investigator using the details at the end of this sheet.

**9. What happens if I change my mind?**

Taking part in this research is voluntary. If you do not want to take part in the research you can withdraw yourself and your data at any time and without giving a reason. If you withdraw from the study your data will be withdrawn from our records. Withdrawing from the study will not affect the standard of care you receive from the healthcare team. If you would like to withdraw at any time we would ask you to let one of the research team know. If you lose the ability to confirm that you are happy to proceed with the study (i.e. if you lose the capacity to consent to ongoing participation), information collected up until this point will be kept.

**10. What if there is a problem?**

If you wish to complain about any aspect of the way in which you have been approached or treated, or how your information is handled during the course of this study, you should contact Dr Kome Gbinigie (Chief Investigator) on 01865 289067 or cuti@phc.ox.ac.uk, or you may contact the University of Oxford Clinical Trials and Research Governance (CTRG) office on 01865 616480, or the head of CTRG, email [ctrg@admin.ox.ac.uk](mailto:ctrg@admin.ox.ac.uk).

The University of Oxford, as Sponsor, has appropriate insurance in place in the unlikely event that you suffer any harm as a direct consequence of your participation in this study.

**11. What will happen to the results of the research study?**

We aim to publish the results so that scientists and doctors are aware of the findings. With your consent we would like to use anonymous quotes from interviews to be included in the research reports. We will also provide your GP practice a copy of our findings for them to display. You will not be personally identifiable in any publication.

**12. Who is organizing and funding the research?**

This study is sponsored by the University of Oxford and the funding for this research comes from the NIHR School for Primary Care (SPCR) and the Wellcome Trust.

**13. Who has reviewed this study?**

All research in the NHS is looked at by an independent group of people, called a Research Ethics Committee, to protect participants’ interests. This study has been reviewed and given favourable opinion by South Central Research Ethics Committee.

**14. Do you have any further questions or concerns?**

If you want to discuss the study please contact Dr Kome Gbinigie, Chief Investigator, by email (cuti@phc.ox.ac.uk) or telephone (01865 289067).

**Thank you for taking the time to read this information leaflet.**
