# Supplementary material for: Does cranberry extract reduce antibiotic use for symptoms of acute uncomplicated urinary tract infections (CUTI)? Protocol for a feasibility study
Source: Trials. 2019 Dec 23;20:767. doi: 10.1186/s13063-019-3860-z (PMC6929469; doi:10.1186/s13063-019-3860-z)
Supplement: Supplementary file 6 — Additional file 6. Informed Consent Form (CUTI interview study). [file 13063_2019_3860_MOESM6_ESM.docx]

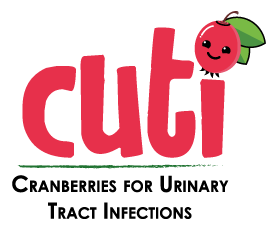
**INFORMED CONSENT FORM – CUTI Interview study**

**Chief Investigator:** Dr Kome Gbinigie. **Address:** Nuffield Department of Primary Care Health Sciences, Radcliffe Primary Care building, Radcliffe Observatory Quarter, Woodstock Road, Oxford, OX2 6GG. **Email:** cuti@phc.ox.ac.uk **Telephone:** 01865 289067­­

**Study Title:** CUTI Interview study **Participant** **ID number: ______________________**

If you are happy to take part in this research then please read each of the statements below and initial the boxes if you agree with them.

**Please initial each box**

1. I confirm that I have read and understood the information sheet version [number], dated [date month year] for the CUTI interview study and have had the opportunity to ask questions and had these answered satisfactorily.

2. I understand that my participation is voluntary and that I am free to withdraw at any time, without giving any reason, and without my medical care or legal rights being affected.

3. I understand that my interview will be audio-recorded and that the interview will be transcribed by an independent person who will transcribe the interview verbatim (word for word).

4. I understand that data collected during the study may be looked at by individuals from the University of Oxford or regulatory authorities, where it is relevant to my taking part in this research. I give permission for these individuals to have access to my records.

5. I understand that the information that I have given to researchers will be transferred to Oxford University and stored securely. I give permission for the research team to use anonymous data collected as part of this study for future research.

6. I give permission for anonymous quotes from interviews to be included in reports of the findings from the research.

7. By signing this consent form, I agree to participate in the CUTI interview study.

**Participant name: ……………………………………. Name of person taking consent:……………………………………………..**

**Date:……………………………………………………….. Date:………………………………………………………………………………………**

**Signature:………………………………………………… Signature:……………………………………………………………………………….**

***When completed, one copy should be given to the participant, one copy should be kept by the research team and one copy should go in the participant’s medical notes***

[
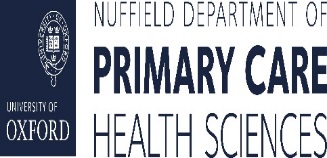
](https://www.google.co.uk/url?sa=i&source=images&cd=&ved=2ahUKEwi0mumojMzcAhVR1xoKHRGQD2IQjRx6BAgBEAU&url=https://www.phc.ox.ac.uk/intranet/communications-engagement/comms/brandguidelines&psig=AOvVaw25IKRdoU5fZKRI-OJ-Rdxv&ust=1533221189259821) [
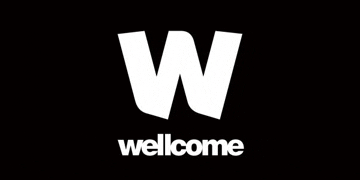
](https://www.google.co.uk/url?sa=i&source=images&cd=&ved=2ahUKEwiuxNPikczcAhWwyYUKHaUYAkAQjRx6BAgBEAU&url=https://jobs.newscientist.com/en-gb/employer/10006940/wellcome-trust/&psig=AOvVaw0mDDZCSW5l1hKxQkGqkRAC&ust=1533222668102409) [
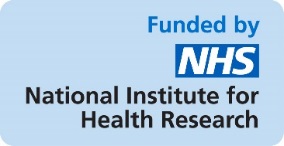
](http://ghrgst.nihr.ac.uk/about-us/)
